# Integrating an Organocatalyst into a Polymeric Gel Framework for the Continuous Microflow Baylis–Hillman Reaction

**DOI:** 10.1021/acsomega.5c09476

**Published:** 2026-02-24

**Authors:** Naresh Killi, Amit Kumar, Leena Nebhani, Franziska Obst, Andreas Richter, Bernhard Reineke Matsudo, Thomas Zentgraf, Dirk Kuckling

**Affiliations:** † Department of Chemistry, Faculty of Science, 26578 Paderborn University, Warburger Str. 100, Paderborn 33098, Germany; ‡ Department of Materials Science and Engineering, 28817 Indian Institute of Technology Delhi, New Delhi 110016, India; § Institute of Semiconductors and Mircosystems, 9169 TU Dresden, Nöthnitzer Str. 64, Dresden 01062, Germany; ∥ Institute for Photonic Quantum Systems, 26578 Paderborn University, Warburger Str. 100, Paderborn 33098, Germany; ⊥ Department of Physics, Faculty of Science, Paderborn University, Warburger Str. 100, Paderborn 33098, Germany

## Abstract

Continuous flow catalysis utilizing gel-bound organocatalysts within a microfluidic reactor represents a compelling strategy in the realm of organic synthesis. In this study, a quinuclidine-based catalytic monomer (QMA) was synthesized to create polymer gel dots through the process of photopolymerization that serve as a support for the catalyst. The resulting gel-bound organocatalysts were assembled within a continuous microfluidic reactor to facilitate the Baylis–Hillman reaction between various aldehydes and acrylonitrile at a temperature of 50 °C. The conversion of the product was assessed using ^1^H NMR spectroscopy as an offline analytical method over a duration of 8 h. The findings indicated that highly reactive aldehydes achieved conversion rates exceeding 90%, in contrast to their less reactive counterparts. Furthermore, these results were juxtaposed with previously published data derived from alternative synthetic methodologies, revealing that the continuous microfluidic reactions employing integrated organocatalysts within polymer networks exhibited significantly higher conversions with reduced reaction times (8 h) at the same temperature (50 °C). Additionally, the influence of different geometries (round, triangular, and square) of the gel dots on catalytic activity was investigated, with round and square gel dots demonstrating slightly superior performance compared with triangular gel dots, attributed to their increased surface area. Moreover, an extended reaction period of 6 days was conducted using 4-bromobenzaldehyde and acrylonitrile, resulting in a conversion rate exceeding 70%, which remained stable for 5 days before experiencing a slight decline due to product accumulation on the gel dots.

## Graphical abstract

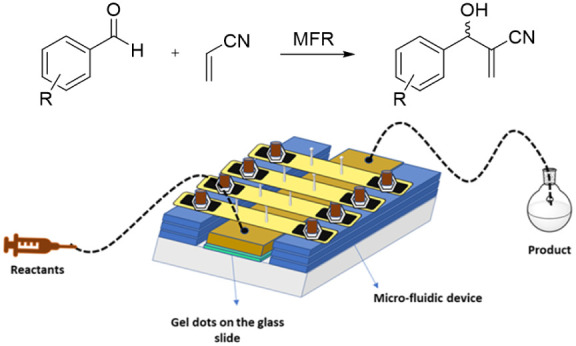

## Introduction

Flow reactors are useful tools to perform continuous organic reactions within the realm of modern chemistry.^1^ In this process, a catalyst is embedded inside a packed bed reactor through which the reactive components are directed via controlled flow managed by pumps. A key advantage of this approach is the ability to reuse the catalyst as its catalytic functionality is fixed at a designated location within the reactor. The continuous flow of reactants not only facilitates the reaction but also aids in the separation of products, thereby eliminating the conventional steps of separation and filtration that are typically required in heterogeneous organic reactions.^2–4^ However, as the reactive materials flow through the packed bed of the catalyst, the accessibility of catalytic sites for the reagents is limited to the surface of the immobilized phase. Flow reactors also enable the parallel monitoring of organic reactions, allowing for real-time adjustments of parameters during the process.^5,6^ These reactors can be miniaturized into smaller units known as microfluidic reactors (MFRs), which offer numerous benefits, including superior heat transfer, laminar flow of reactants, and precise control over reaction conditions.^7^ Additionally, microfluidic reactors facilitate safer handling and rapid analysis of a variety of reactions.^8^ They can be integrated with various monitoring techniques, such as nuclear magnetic resonance spectroscopy (NMR), high-performance liquid chromatography (HPLC), infrared (IR), and ultraviolet/visible (UV/vis) spectroscopy, as well as mass spectrometry.^9–13^ Furthermore, microfluidic reactors are deemed safer, as they allow reactions to be conducted with minimal quantities of reactants, enabling the use of highly reactive or even toxic substances.^14^

In our approach, the catalytic functionalities are immobilized within cross-linked polymeric networks, which not only supports their reusability but also ensures long-term activity.^15,16^ The swelling of the polymer in the presence of solvents enhances the accessibility of catalytic sites embedded within the bulk of the polymer, in contrast to solid catalysts that only provide access to surface sites.^11,17^ The application of polymer gels with a high cross-linker content, embedded with catalysts and enzymes for microfluidic reactions, has already been demonstrated. Hexagonal frameworks of polymer gels adhered to glass slides have been fabricated using photolithography and utilized for a variety of reactions in MFR studies.^13,16,18–21^ In this context, the immobilization of amine derivatives as catalysts within surface-bound polymer gels has been explored for conducting Knoevenagel reactions involving various aldehydes and malononitrile.^16,18^ Additionally, the immobilization of *L*-proline derivatives has been reported for catalyzing the synthesis of aromatic azoxy compounds through the dimerization of nitrosobenzene derivatives under mild conditions.^19^ Similarly, *L*-proline derivatives bound to polymer gel dots have been utilized for asymmetric aldol reactions.^20^

The Baylis–Hillman reaction, which involves the coupling of an electron-deficient olefin with a carbon electrophile such as an aldehyde, ketone, or imine, is typically catalyzed by tertiary amines.^22–24^ However, studies on catalyzing the Baylis–Hillman reaction in continuous flow systems have been limited. Acke and Stevens investigated the reaction between 4-nitrobenzaldehyde and methyl acrylate in a plug-flow microreactor setup, observing a 30% time reduction to complete the reaction.^25^ The Baxendale group reported the reaction between isobutyraldehyde and acrylonitrile in continuously stirred tank reactors, achieving an impressive throughput of over 200 mmol/h at 65 °C by aligning six 100 mL reactors in series.^26^ Ishitani et al. developed a packed bed reactor containing 4-(dimethylamino)­pyridine (DMAP) immobilized on silica for the reaction between β-nitrostyrene and ethyl glyoxalate in toluene at room temperature, noting a decline in conversion rates to approximately 50% within 48 h due to catalyst degradation.^27^

To date, the catalysis of the Baylis–Hillman reaction using microfluidic technology with an immobilized catalytic system in a polymer gel has not been explored. Considering this, we propose the fabrication of polymeric gel dots incorporating quinuclidine-based tertiary amine moieties via photolithography and their integration into a microfluidic chip. These gels serve as catalysts for the Baylis–Hillman reaction, facilitating the transformation of various aldehydes, selected as carbon electrophiles, with acrylonitrile as the activated olefin. The reaction was performed and optimized using a microfluidic system, where the chamber was packed with immobilized tertiary amine catalytic sites within polymer gel dots arranged in a hexagonal array. The catalytic activity was evaluated, and continuous-flow reactions were conducted by using this microfluidic reactor. A schematic representation of the reactor system assembly is provided in Figure 1.

**Figure 1:**
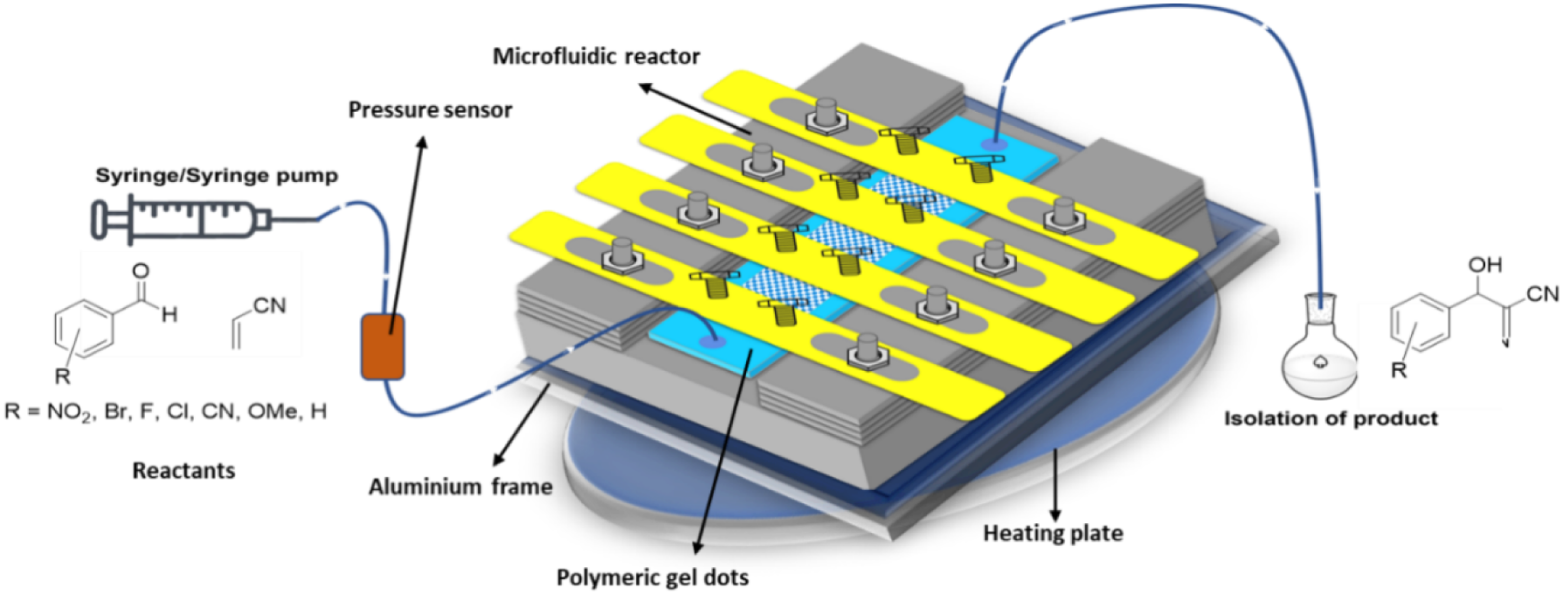
Graphical representation of the microfluidic reactor system assembly.

## Experimental Section

### Fabrication of Polymer Gel Dots on Methacrylate-Anchored Glass Substrates

Polymer gel dots were fabricated on modified glass substrates via photolithography, following a previously reported procedure.^16^ The gel composition consisted of 90% catalytic monomer (QMA), 5% gelling agent (MMA), and 5% cross-linker (EGDMA), as detailed in Table 1. Photopolymerization was carried out using an Omnicure S1500 UV lamp (Lumen Dynamics), with UV irradiation applied at an intensity of 1.28 W at the end of the lighting cable, positioned 8 cm from the assembly. A total of 15 mmol of monomers (QMA, MMA, and EGDMA) were dissolved in 1.7 mL of an ethanol:water mixture (v/v 7:3), with an initiator concentration of 33.3 mg/mL. From this solution, 850 μL was transferred into an incubation chamber gasket made of black polypropylene to minimize scattered radiation. A methacrylate-functionalized glass substrate was placed over the gasket, and a photomask—designed to allow UV transmission through a diamond-shaped array of 662 dots—was positioned atop the modified glass. The assembly was exposed to UV radiation, ensuring uniform irradiation over the patterned array. Photolithography enabled the fabrication of polymer gel dots with various geometries (spherical, trigonal, and square), using corresponding photomasks while maintaining a constant array volume (6.37 μL) and dot count (662). A schematic representation of the photolithographic process is shown in Figure 2. Following UV exposure, the assembly was dismantled, and a glass substrate with covalently bound polymer gel dots was obtained. The substrate was sequentially washed with water and isopropanol and then air-dried at room temperature before use in catalytic studies.

**Table 1:** Fabrication of Polymer Gel Dots with Various Compositions and Irradiation Parameters

Code	Catalyst	Gelling agent	Cross-linker	UV intensity (W)	Irradiation time (s)	Observation
A	QMA (90%)	MMA (9%)	EGDMA (1%)	1.2	100	No gel formation
B	QMA (90%)	MMA (5%)	EGDMA (5%)	1.2	100	Gel dots with film formation in nonexposed area
C	QMA (90%)	MMA (5%)	EGDMA (5%)	1.2	90	Stable gels

**Figure 2:**
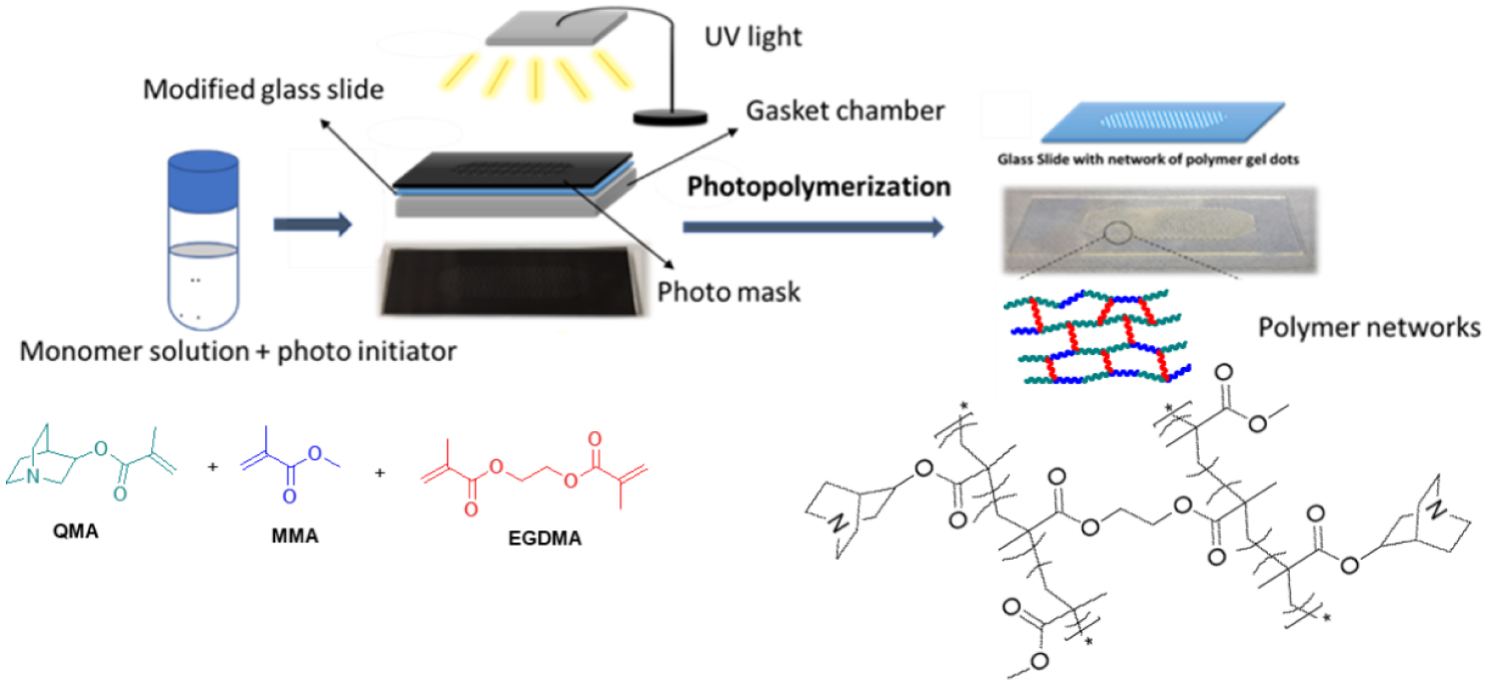
Fabrication process of polymer gel dots by using photolithography.

### Procedure for Setting Up the Microfluidic Reactor Assembly

The microfluidic reactor was assembled following a previously reported design.^13^ A pictorial representation of the self-assembled microfluidic system is shown in Figure S2. The reactor consists of a compartmentalized PTFE layer with an imprinted chamber structure (250 μm in height) designed to accommodate polymer gel dots and a glass substrate functionalized with a diamond-shaped array of gel dots. The two components were securely fixed in an alumina holder by using screws tightened to a torque of 0.8 N m. To prevent leakage, Parafilm was applied along the edges of the reaction chamber before connecting the reactor to inlet and outlet PTFE capillary tubes (i.d. = 0.2 mm, Fisher Scientific).

The inlet tube was attached to a 10 mL syringe (Hamilton 1000 series) containing the reactant solution, which was mounted on a syringe pump (Legato 200, KD Scientific) to regulate the flow rate. A microfluidic inline pressure unit (Fluigent) was installed on the inlet tube between the syringe and the reactor to monitor the back pressure. The outlet tube was directed into a collection vial. The assembled reactor was positioned on an aluminum holder atop a heating plate, with a thermometer placed to monitor the reaction temperature.

### Procedure to Perform Flow Reactions in the Microfluidic Reactor

All the microfluidic reactions were carried out at 50 °C. Initially, the polymeric gel dots were preswollen in the reaction mixture, ethanol:water (v/v = 7:3), at a flow rate of 2.0 μL/min for 2 h. Subsequently, the syringe was loaded with the respective reactant solution, and the chemicals were passed through the microfluidic reactor, with a flow rate of 0.5 μL/min. The product was collected at the receiving end of the reactor over 8 h, excluding an initial equilibration time of 3.5 h. The reaction conversion was determined as the average yield of the collected product.

### Determination of Reaction Conversion

Off-line ^1^H NMR spectroscopy was used for the determination of reaction conversion. 100 μL of the collected product solution was taken in an NMR tube, and the solution was diluted with 500 μL of DMSO-*d*_6_. The samples were mixed gently before measurement. The conversion of the reaction was estimated using the integral of the product peak with the respective integral of the aldehyde peak.

## Results and Discussion

The catalytic monomer quinuclidin-3-yl methacrylate (QMA) was synthesized via nucleophilic substitution, wherein the hydroxyl group of quinuclidin-3-ol reacted with methacryloyl chloride in the presence of triethylamine as a base (Scheme 1).^29^ More details can be found in the SI.

**Scheme 1:**
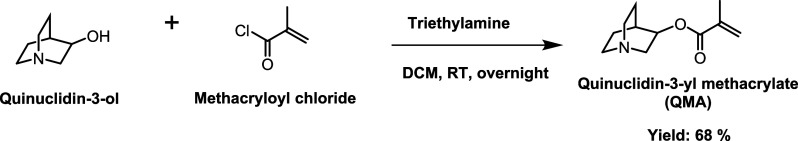
Reaction Scheme Showing the Synthesis of Catalytic Monomer QMA

To enable the fabrication of polymer gel dots on conventional microscopic glass substrates (Figure 2), surface modification was carried out by using methacrylate functionalization. This modification enabled a covalent attachment of the gel dots onto the glass surface, enhancing adhesion to the substrate and preventing detachment during purification and flow reactions.

Polymer gel dots were synthesized via UV-assisted photopolymerization of monomeric components. Various formulations were prepared by adjusting the relative monomer ratios (total monomer concentration: 8.8 mol L^–1^) and varying UV irradiation times, as detailed in Table 1. The catalytic monomer (QMA) concentration was fixed at 90%, while the complementary monomer (MMA) and cross-linker (EGDMA) were systematically varied. Formulations with lower cross-linking density exhibited poor stability, while increasing cross-linking improved gel integrity. Additionally, UV exposure time played a critical role in gel formation, with an optimized irradiation duration of 90 s at an intensity of 1.2 W yielding well-defined gel dots without polymerization on nonexposed areas.

Following optimization, polymer gel composition C was selected for further studies based on its superior stability and quality. This composition was then used to fabricate gel dots with different geometries—round, triangular, and square—using respective photomasks (Figure 3). The influence of the gel geometry on catalytic performance was investigated in a microfluidic reactor system. The photomask design maintained a constant array volume (6.37 μL), top surface area (63.69 mm^2^), and total polymer dot count (662), ensuring controlled comparison across geometries (Table S1).

**Figure 3:**
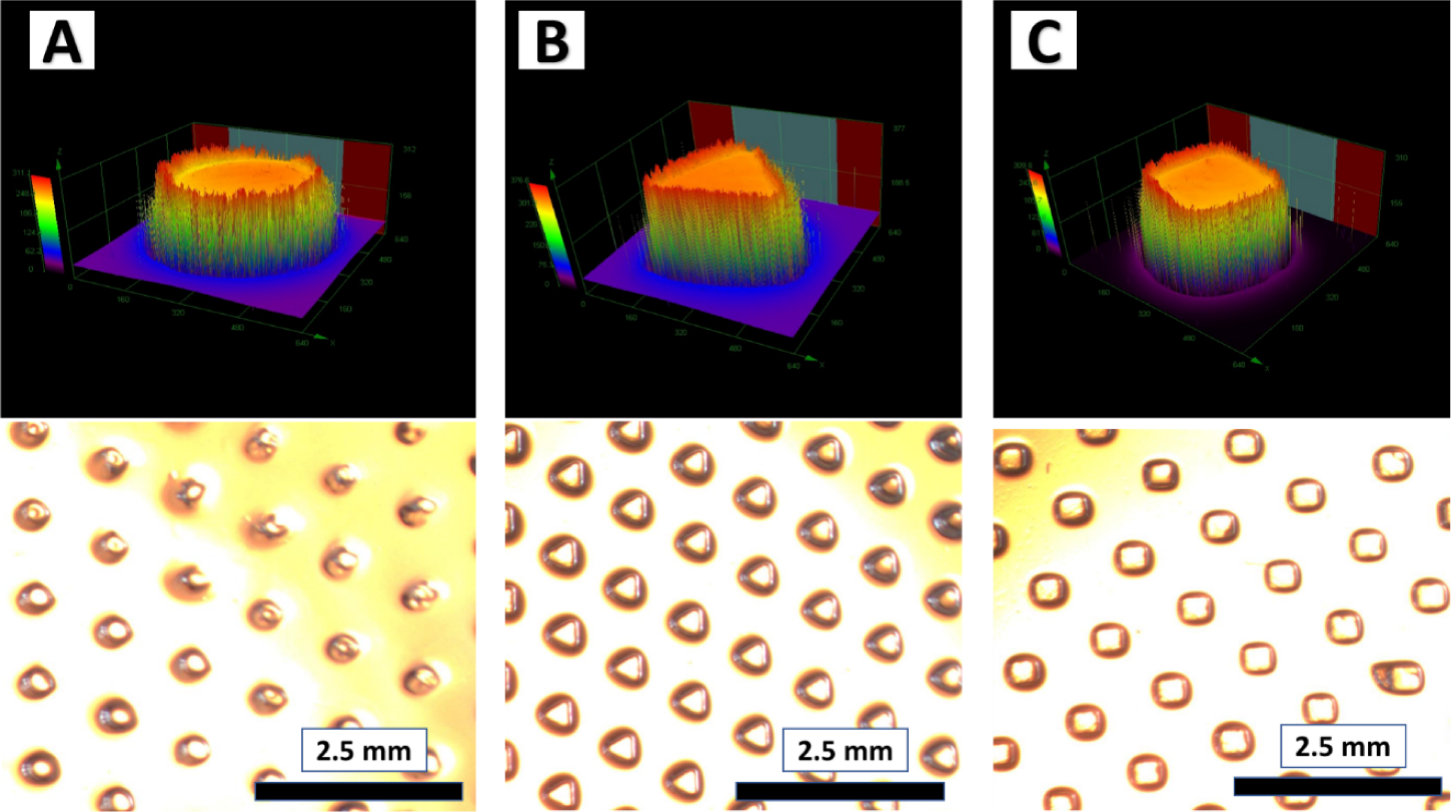
Microscopic images of polymer gel dots with various geometries, A) round, B) triangular, and C) square. The top images were captured by a 3D measuring laser microscope (LEXT), and the bottom images were captured by a white-light microscope.

Polymer networks with different geometries were fabricated by using photolithography under the conditions specified in Table 2. The resulting gel structures were analyzed using white light microscopy and a 3D measuring laser microscope (LEXT) (Table S2). The total surface areas of the 662 gels were approximate values of 245 mm^2^ (round), 183 mm^2^ (triangular), and 282 mm^2^ (square) (Table 2). The surface area of the gels was higher compared to the theoretical values (photomask) due to the height and width of the gel dots. These polymer gel dots were subsequently employed in continuous microfluidic Baylis–Hillman reactions to assess the impact of gel geometry on catalytic activity under flow conditions.

**Table 2:** Continuous Microfluidic Reaction between 4-Nitrobenzaldehyde and Acrylonitrile Using Different Geometries of Gel Dots

					Pressure (mbar)	
Composition	Geometry of gel dots	Surface area (mm^2^)^a^	Total surface area of all the gels (662) (mm^2^)	Conversion (%)	Preswelling of gel dots at flow rate of 2 μL/min	Reaction process at flow rate of 0.5 μL/min
C	Round	0.37	245	100	8–17	1–10
C	Triangle	0.28	183	90	4–14	1–5
C	Square	0.43	282	100	8–17	1–9

a The values were calculated from the LEXT data of the single gel (Table S2).

Prior to conducting microfluidic reactions, the swelling properties of the polymeric gels were evaluated in the reaction solvent mixture (ethanol:water, v/v = 7:3), as organocatalytic activity is significantly influenced by gel swelling.^16,30^ During this process, the polymeric network absorbs the solvent, causing polymer chains to expand and form a highly porous structure, thereby increasing the accessibility of the catalytic sites. To assess the swelling behavior, monomeric solutions were introduced into Pasteur pipet tips and polymerized under UV irradiation following the parameters outlined in Table 1. The resulting gels (macrogels) were extracted, purified according to the standard gel dot preparation protocol, and subsequently dried at room temperature to form xerogels. Approximately 25 mg of dried xerogel (with an initial volume of ∼23 mm^3^ (assuming a cylindrical shape) (Figure 4A) was placed in sample vials in duplicate and swollen in the reaction solvent mixture overnight. The swollen gels (Figure 4B) exhibited an increase in weight (∼123 mg) and volume (∼147 mm^3^), corresponding to a swelling ratio of approximately 5-fold by weight and 6-fold by volume, as determined using (eqs S1 and S2 Table S1). These results confirmed that the selected solvent system was appropriate for further catalytic studies. Notably, while all gels exhibited similar swelling behavior, the volumetric expansion of gel dots was significantly lower compared to that of macrogels due to their surface attachment. Swelling properties of the gel dots with various geometries (round, trigonal, square) were performed for 2 h, and the optical microscopic images of the gel dots are shown in Figure S3.

**Figure 4:**
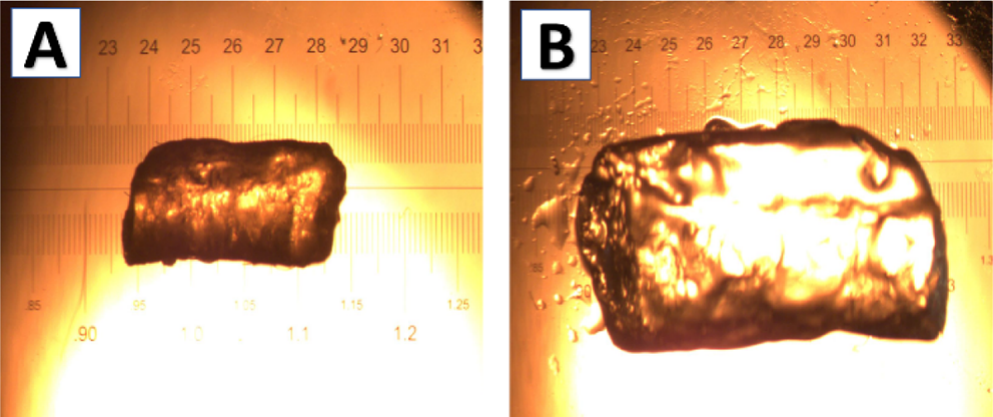
Swelling studies of the gels: A) dry (xero) gel and B) swollen gel.

The fabricated polymer gel dots were employed as catalysts for continuous microfluidic Baylis–Hillman reactions involving various aldehydes and acrylonitrile within an assembled microfluidic reactor. The reaction setup is depicted in Figure S2. A hexagonal-shaped array of 662 polymer gel dots was positioned within a PTFE-based reaction chamber. The assembled system was secured in an aluminum holder, with the reactor connected to a syringe via PTFE capillary tubes (i.d. = 0.2 mm) for reactant introduction, while an outlet tube directed the product to a collection vial. Additionally, a pressure sensor was integrated into the inlet tube to monitor the backpressure within the reactor.

The microfluidic reaction was conducted at 50 °C, and the optimization of the microfluidic reaction conditions and residence time was studied according to our previously reported work.^20^ Initially, the polymer gel dots were preswollen in the reaction solvent mixture (ethanol:water, v/v = 7:3) for 2 h at a flow rate of 2 μL/min. This swelling process allowed polymer network expansion, increasing the accessibility of catalytic sites, as described in the swelling studies. Following the swelling phase, the syringe was loaded with a reactant solution containing aldehyde and acrylonitrile in a 1:2 molar ratio (0.04 mmol of aldehyde and 0.08 mmol of acrylonitrile in 1 mL of ethanol:water, v/v = 7:3) and introduced into the microfluidic reactor at a flow rate of 0.5 μL/min while maintaining a temperature of 50 °C.

Reaction conversion was determined via offline ^1^H NMR spectroscopy after the eluent was collected for 8 h, excluding the initial 3.5 h equilibration time. A 100 μL aliquot of the collected solution was mixed with 400 μL of DMSO-*d*_6_ for NMR analysis. Conversion percentages were calculated based on the integration values of the aldehyde proton (δ = 10.2 ppm) and the product peak (∼δ = 5.5 ppm), as shown in Figures S4–S12. The results indicated that highly reactive aldehydes, such as 2-nitrobenzaldehyde, 3-nitrobenzaldehyde, 4-nitrobenzaldehyde, 4-cyanobenzaldehyde, and 4-bromobenzaldehyde, achieved conversions of 83–100%. In contrast, less reactive aldehydes, including benzaldehyde and 4-methoxybenzaldehyde, exhibited significantly lower conversions (16–23%), as summarized in Table 3.

**Table 3:**
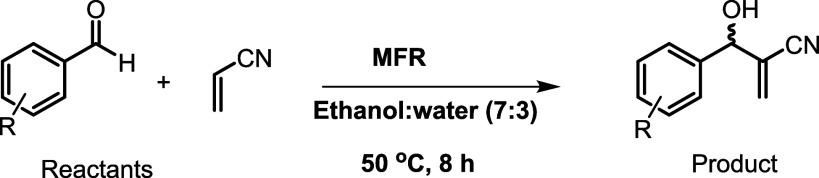

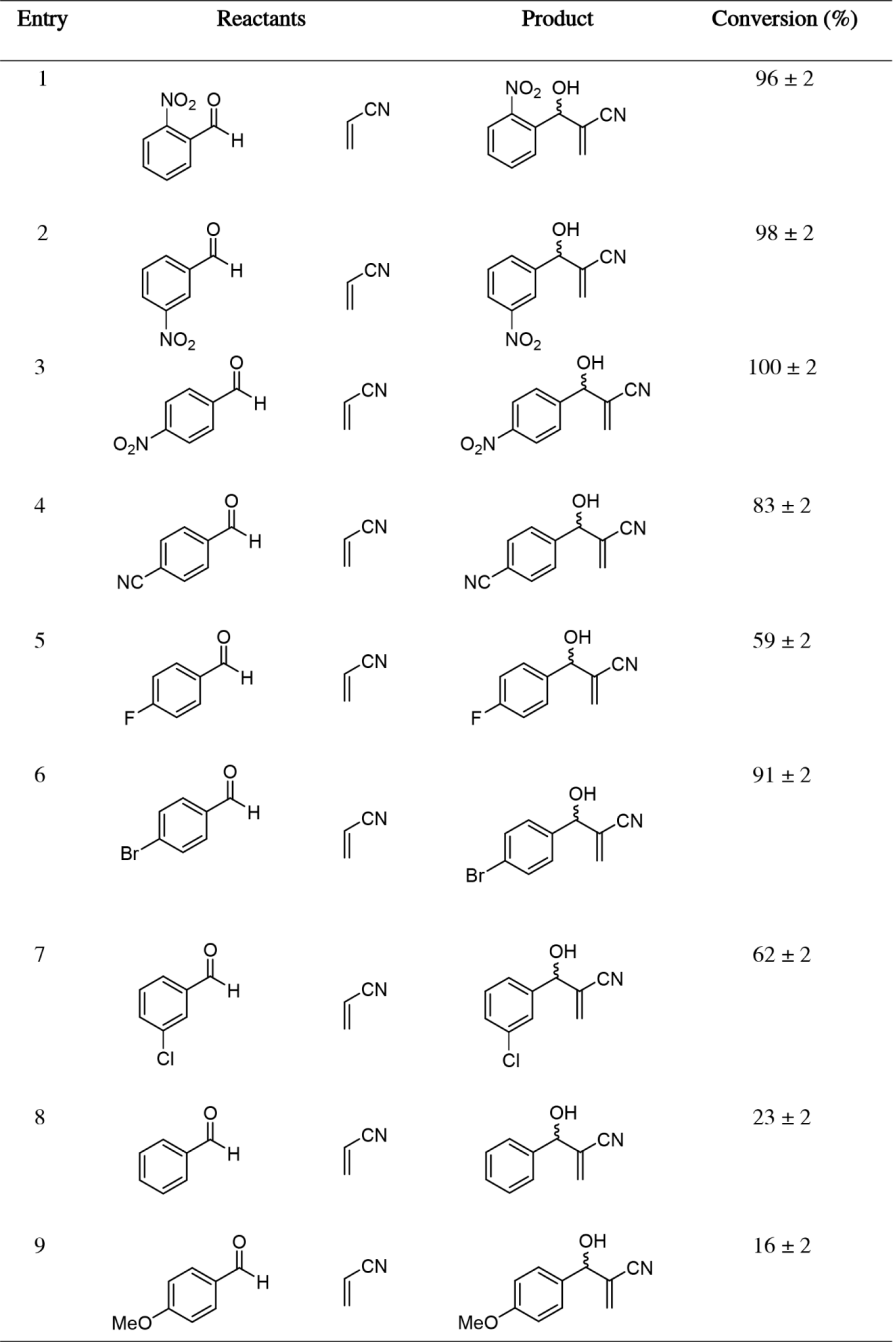
Baylis–Hillman Reaction between Various Aldehydes and Acrylonitrile Quantified Using Offline ^1^H NMR Analysis

Furthermore, these conversion efficiencies were markedly superior to those observed in conventional batch reactions employing quinuclidine-based polymeric networks as catalysts.^28,29^ For example, entries 1, 2, 4, and 6 in the MFR demonstrated conversion rates of 96%, 98%, 83%, and 91%, respectively, which were approximately 26%, 50%, 40%, and 51% higher than their batch counterparts. Notably, the average conversion in MFR reactions was determined after only 8 h (following the 3.5 h equilibration time), whereas batch reactions required 16 h to reach their final conversions. These findings underscore the superior efficiency of the microfluidic reactor system, where reactions proceed at an accelerated rate with enhanced catalytic turnover compared with batch methods. Additionally, the continuous nature of the process ensures a consistently higher turnover number, as catalysis is sustained using a fixed amount of catalyst, which remains reusable for multiple reaction cycles. The comparison studies of the MFR were performed with the same catalyst (quinuclidine) as the major composition in the polymer networks in batch conditions to understand the efficiency of the catalyst in MFR as compared to batch reactions.

The catalytic performance of polymer gel dots with different geometries (round, triangular, and square) was evaluated in a microfluidic reactor for the continuous reaction between 4-nitrobenzaldehyde and acrylonitrile. The reaction conversion and pressure variations are summarized in Table 2. Round and square gel dots achieved complete conversion (100%), whereas triangular gel dots exhibited a slightly lower conversion (90%). During the preswelling phase at a flow rate of 2 μL/min, the reactor pressure ranged between 4–17 mbar, which subsequently stabilized at 1–10 mbar during the reaction at 0.5 μL/min. These findings indicate that round and square gel dots provide enhanced catalytic activity compared to triangular ones, likely due to their larger surface areas (245 mm^2^ for round, 183 mm^2^ for triangular, and 282 mm^2^ for square gel dots) (Table 2).

A long-term microfluidic reaction between 4-bromobenzaldehyde and acrylonitrile was performed over 6 days to evaluate the efficiency of the continuous process. ^1^H NMR spectra were recorded every 24 h (Figure 5A), and the reaction conversion remained between 70–80% for the first 5 d before slightly decreasing to 66%, likely due to product accumulation on the polymer network, decreasing the accessibility of the catalyst. Moreover, the flow of the reaction was slightly decreased, indicating a blocking of the continuous process. This was clearly observed by the change in the color of the gel dots, which was similar to the product after the reaction. However, optical inspection after the reaction did not show any detachment of the gel structures. Figure 5B shows the conversion as a function of the reaction time. On average, the continuous flow reactions maintained 76% conversion over 6 days, corresponding to a turnover frequency (TOF) of approximately 0.74 h^–1^ based on the residence time of 3.5 h, whereas the corresponding batch reactions carried out for 20 h yielded a lower TOF of 0.17 h^–1^. These results clearly indicate the superior catalytic efficiency of the continuous microfluidic reaction system compared to that of conventional batch methods. In flow reactions, the interaction time between reactants and the catalyst is much shorter than in the batch method. These findings highlight the advantages of continuous microfluidic catalysis, including sustained activity, higher turnover efficiency, and improved process stability over extended operation times. Further, the continuous flow approach enables the use of a fixed, minimal catalyst amount to process a large reaction volume with the added advantage of catalyst reusability.

**Figure 5:**
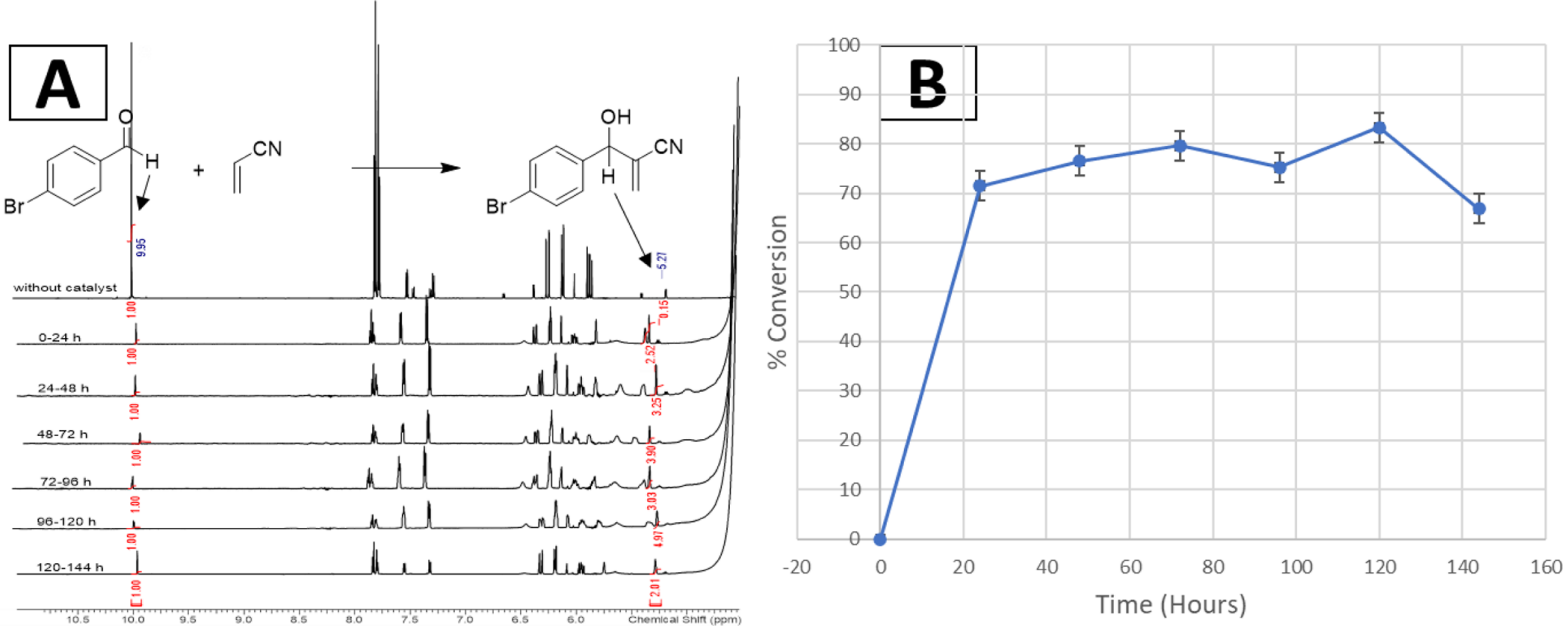
Long-time microfluidic reaction between 4-bromobenzaldehyde and acrylonitrile, A) ^1^H NMR spectra at different time intervals, and B) graphical representation of conversion (%) vs time (h).

## Conclusions

In this study, we successfully fabricated polymeric networks based on the catalytic monomer quinuclidin-3-yl methacrylate (QMA) via photolithography to generate gel dots with distinct geometries (round, triangular, and square). The gel composition consisted of 90% QMA, 5% methyl methacrylate (MMA) as a complementary monomer, and 5% ethylene glycol dimethacrylate (EGDMA) as a cross-linker. The resulting gels exhibited stability and were employed in a microfluidic reactor to evaluate their catalytic efficiency in the Baylis–Hillman reaction between various aldehydes and acrylonitrile at 50 °C.

The catalytic activity of the gel dots in continuous flow conditions was determined via NMR spectroscopy, revealing conversion rates of 90–100% for highly reactive aldehydes such as nitrobenzaldehydes, 4-formylphenyl nitrile, and 4-bromobenzaldehyde, with reduced efficiency observed for less reactive aldehydes. Continuous flow reactions demonstrated enhanced reaction rates and higher catalytic turnover compared to conventional batch methods, underscoring the superior efficiency of the flow-based process. Among the different geometries, round- and square-shaped gel dots exhibited the highest catalytic activity (100%), outperforming triangular gel dots (90%) due to their larger surface area. These results suggest that geometric optimization is an important factor in microfluidic reactors. Additionally, a long-term reaction between 4-bromobenzaldehyde and acrylonitrile was conducted to assess catalytic stability under continuous conditions. Over a period of 5 d, catalytic activity remained between 70–80%, with a slight decline attributed to product accumulation within the reactor. These findings highlight the potential of QMA-based polymeric gel dots as highly efficient organocatalysts in continuous microfluidic systems, paving the way for more sustainable and scalable catalytic processes in organic synthesis.

## Supplementary Material



## Notes

Materials, characterization, and synthesis of catalytically active monomer (QMA), including ^1^H NMR spectra; photographs of the experimental setup; swelling studies of the gels; calculations for surface area; synthesis of Baylis–Hillman products, including ^1^H NMR spectra for all compounds (PDF)
