# Supplementary material for: Integrating an Organocatalyst into a Polymeric Gel Framework for the Continuous Microflow Baylis–Hillman Reaction
Source: ACS Omega. 2026 Feb 24;11(9):14448–55. doi: 10.1021/acsomega.5c09476 (PMC12980247; doi:10.1021/acsomega.5c09476)
Supplement: Supplementary file 1 [file ao5c09476_si_001.pdf]

Supporting information

Integrating an organo-catalyst into a polymeric gel framework for the continuous micro-flow Baylis-Hillman reaction

Naresh Killi[1], Amit Kumar[2], Leena Nebhani[2], Franziska Obst[3], Andreas Richter[3], Bernhard Reineke Matsudo[4], Thomas Zentgraf[4,5] and Dirk Kuckling[1]*

[1] Department of Chemistry, Faculty of Science, Paderborn University, Warburger Str. 100, 33098 Paderborn, Germany

[2] Department of Materials Science and Engineering, Indian Institute of Technology Delhi, New Delhi, 110016, India

[3] Institute of Semiconductors and Mircosystems, TU Dresden, Nöthnitzer Str. 64 01062 Dresden, Germany

[4] Institute for Photonic Quantum Systems, Paderborn University, Warburger Str. 100, 33098 Paderborn, Germany

[5] Department of Physics, Faculty of Science, Paderborn University, Warburger Str. 100, 33098 Paderborn, Germany

* Correspondence: dirk.kuckling@uni-paderborn.de

Materials. Methacryloyl chloride (96 %), 3-quinulidinol (99 %), methyl methacrylate (MMA) (98 %), 4-methoxy benzaldehyde (98 %), 3-(trichlorosilyl)propylmethacrylate (> 90 %), 4-cyanobenzaldehyde (95 %), 4-fluorobenzaldehyde (98 %) and triethylamine (99 %) were purchased from Sigma Aldrich. Benzaldehyde (98 %), 2-nitrobenzaldehyde (99 %) and 3-nitrobenzaldehyde (98 %) were purchased from Tokyo Chemical Industries (TCI). Acrylonitrile (99 %) and 3-chlorobenzaldehyde (99 %) were obtained from Acros Organic. Ethylene glycol dimethacrylate (EGDMA) (97.5 %) and 4-nitrobenyaldehyde (98 %) were obtained from Merck. 4-Bromobenzaldehyde (99 %) was obtained from Alfa Aesar. Aqueous ammonia (25 %), hydrogen peroxide (30 %), ethanol (technical grade), dichloromethane (DCM) (HPLC), sodium chloride, and aluminium oxide were obtained from Stockmeier Chemie, Bielefeld. Sodium hydrogen carbonate and sodium sulfate were procured from VWR International GmbH Chemicals, Langenfeld. Argon 5.0, was obtained from Wohning Gas GmbH, Paderborn. Lithium phenyl-2,4,6-trimethylbenzoyl-phosphinate (LPTMBP) was used as an initiator and was synthesized as per reported procedure.[28] Methacryloyl chloride was used for the synthesis after distillation.

Characterizations. ^{1}H and ^{13}C NMR (Nuclear Magnetic Resonance) spectra of all samples were recorded on a Bruker "Ascent 700" spectrometer using deuterated solvents. NMR signals were recorded with respect to the reference, tetramethyl silane (TMS) signal. The ESI-MS measurements were performed out on a "Synapt-G2 HDMS" mass spectrometer from Waters in combination with a TOF analyzer. The images of the gels were captured by a light microscope from Hund Wetzlar with a cold light source FLQ 150 M. The images were captured with a camara "iDSuEye", and uEye Cockpit (software). The diameters of the gels were measured using a microscopic ruler from PYSER-SGI Ltd. A LEXT 3D Measuring Laser Microscope OLS4000 of *Olympus* was used for the recording of 3D images of gel dots and the

measurement of their size. Following acquisition parameters were adjusted with scanning mode (XZY fast scan), Image size (1024 x 1024 Pixels), objective lens (MPLAPONLEXT20x).

Synthesis of quinuclidin-3-yl methacrylate (QMA). 3-Quinuclidinol (6 g, 47.5 mmol) was put in 100 mL two necked round bottom flask and dissolved in anhydrous dichloromethane (75 mL). Triethylamine (9.6 mL, 71.4 mmol) was added to the reaction mixture then stirred for 30 min at room temperature under argon atmosphere. Further, the reaction mixture was cooled down to 0 °C using an ice bath and methacryloyl chloride (5.6 mL, 57.1 mmol) was added dropwise over the period of 20 min with continuous stirring. The contents were stirred at room temperature overnight. The reaction was quenched with water and then the organic fraction was washed with water, followed by saturated $NaHCO_3$, ammonia solution, and brine. The organic layer was dried over anhydrous sodium sulphate. The crude product was obtained after removal of solvent and further purified by vacuum distillation to obtain monomer designated as QMA in 68 % yield.

^{1}H NMR (500 MHz, $CDCl_3$, 303 K) δ (ppm) = 6.09 (dd, J = 1.0, 1.7 Hz, 1H), 5.54 (quin, J = 1.6 Hz, 1H), 4.82 (dt, J = 1.2, 4.2 Hz, 1H), 3.24 (ddd, J = 2.2, 8.3, 14.7 Hz, 1H), 2.92 - 2.81 (m, 2H), 2.81 - 2.67 (m, 3H), 2.04 - 1.99 (m, 1H), 1.94 - 1.92 (m, 3H), 1.84 (br d, J = 1.9 Hz, 1H), 1.70 - 1.63 (m, 1H), 1.59 - 1.51 (m, 1H).

^{13}C NMR (176 MHz, $CDCl_3$, 298 K) δ (ppm) = 167.0, 136.4, 125.3, 71.3, 55.4, 47.3, 46.3, 25.1, 24.4, 19.6, 18.2.

ESI-MS (m/z): mass calculated: 196.1338 Da, mass found: 196.1351 Da, $C_{11}H_{18}NO_2^+$ $[M + H]^+$

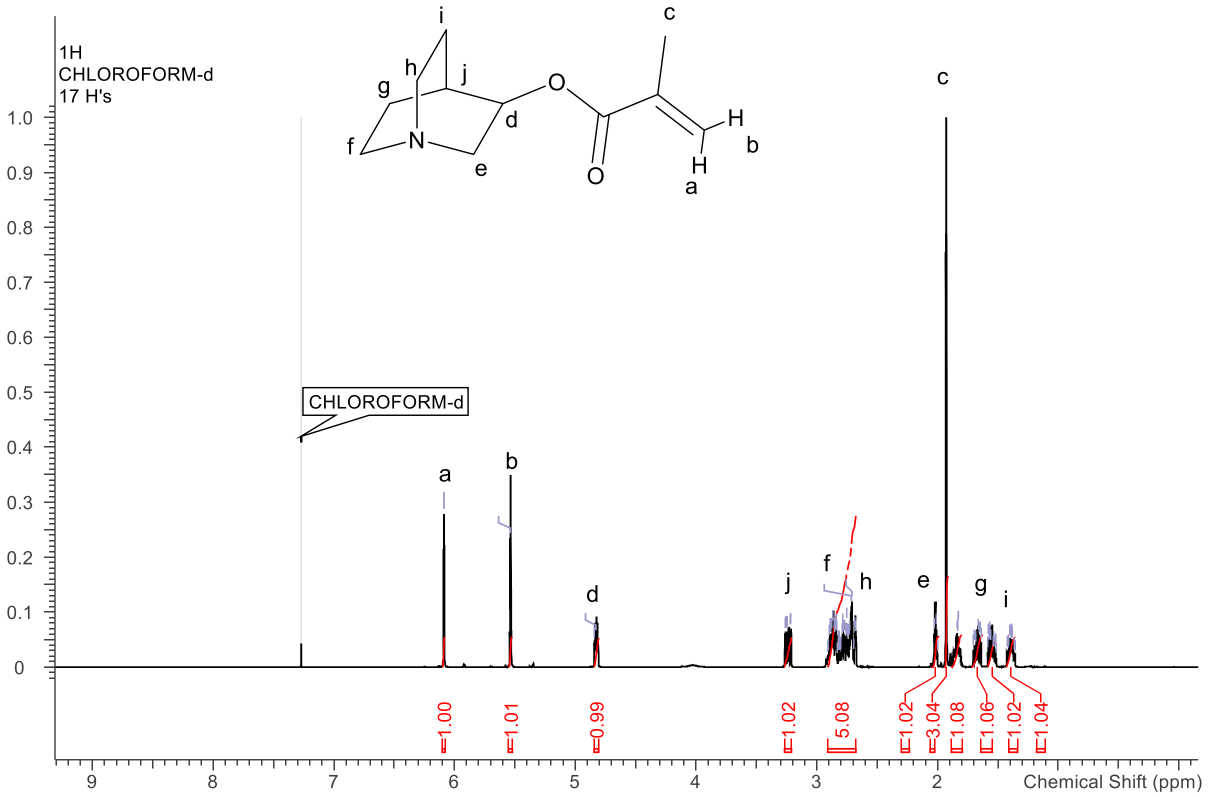

Figure S1. ^{1}H NMR spectra of quinuclidin-3-yl methacrylate (QMA)

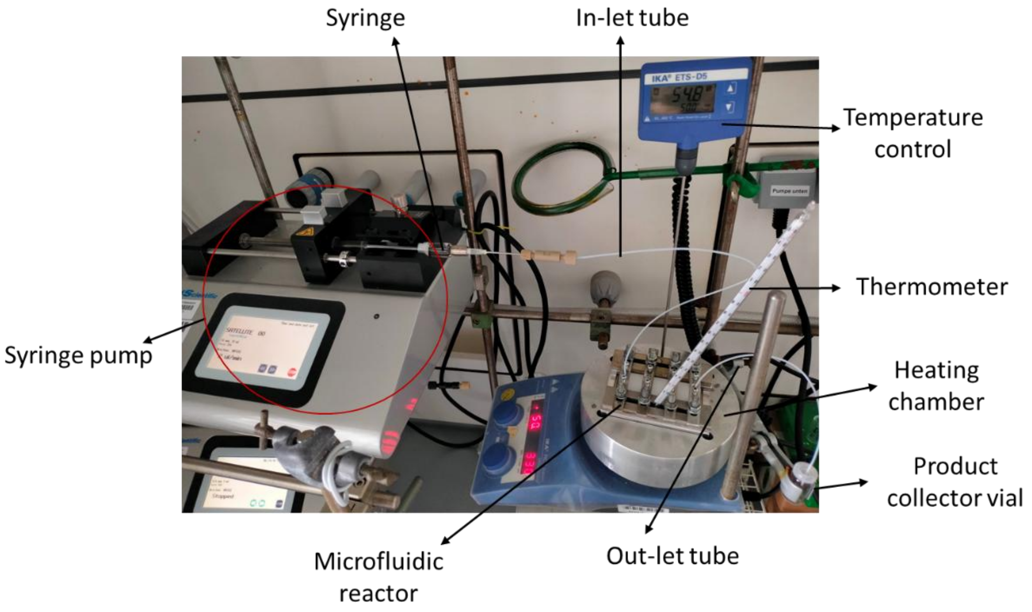

Figure S2. Pictorial representation of the microfluidic reactor system assembly

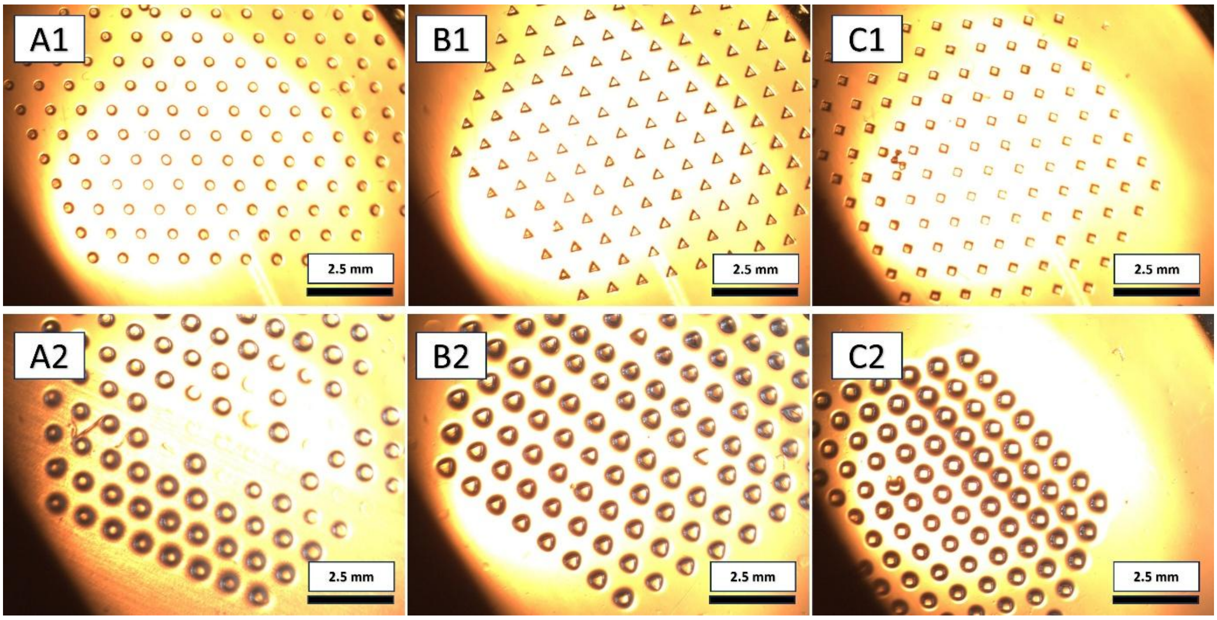

Figure S3. Microscopic images of polymer gel dots with various geometries, A) round, B) triangular and C) square. The top images, A1, B1, C1 are before swelling and the bottom images, A2, B2, C2 are after swelling gel dots.

Swelling studies of gel dots. The swelling behavior of the optimized polymer gel dot compositions was evaluated by measuring solvent uptake and volume changes before and after immersion in the reaction solvent mixture (ethanol:water, v/v 7:3). Briefly, 0.2 mL of the monomer solution was introduced into a Pasteur pipette and polymerized under UV irradiation, following the parameters outlined in Table 1. After polymerization, the cylindrical gels were extracted from the pipette, thoroughly washed with water and 2-propanol overnight, and dried at room temperature until a stable weight was achieved.

The resulting xerogels were subsequently swollen in the respective solvent mixtures overnight. The average weights of the gels before and after swelling were recorded to determine solvent uptake. The percentage of solvent uptake was calculated using Equation S1.

$$Percentage\ of\ solvent\ uptake = \frac{Wt.Sg - Wt.Xg}{Wt.Xg}\ 100 \qquad \text{(S1)}$$

Where, Wt.Sg = Weight of the swollen gel (mg), and Wt.Xg = Weight of the xerogel (mg)

The volume of the cylindrical gels before and after swelling was determined using Equation 2. Gel dimensions, including length and width, were measured using a microscopic ruler and are presented in optical images (Figure 4). The swelling ratio was then calculated using Equation S2.

$$Degree\ of\ swelling\ (Ds) = \frac{Vs}{Vx} \qquad \text{(S2)}$$

Where, Vs = Volume of the swollen gel (mm^3), and Vx = Volume of the xerogel (mm^3),

Volume of the cylindrical gel was calculated by following equation, S3.

$$V = \pi\, r^2\, h \qquad \text{(S3)}$$

Where, V = Volume of the gel (mm^3), r = Radius of cylindrical gel (mm), and h = height of the gel (mm)

Swelling studies of the gels:

Table S1. Swelling properties of the gel composition

No.	Gel composition	Xerogel weight (mg)	Swollen gel weight (mg)	Xerogel		Swollen gel	
				Width (mm)	Length (mm)	Width (mm)	Length (mm)
1	QMA90MMA5EGDMA5	24.0	119.2	2.5	4.5	4.7	8.0
2	QMA90MMA5EGDMA5	26.7	126.0	2.5	5.0	4.7	9.0
	Average	25.35	122.6	2.5	4.75	4.7	8.5

Volumes were calculated by taking respective approximations (cylinder) into account.

Volume of Xerogel (Vx) $= \pi r^2 h$ with r = 1.25 mm and h = 4.75 mm

$= 23.3\ mm^3$

Volume of Swollen gel (Vs) $= \pi r^2 h$ with r = 2.35 mm and h = 8.5 mm

$= 147.5\ mm^3$

Calculations for surface area:

Table S2: Measurement of different geometry of gel dots using LEXT laser microscope

No.	Geometry of the gel dots	Width (µm)	Height (µm)
1	round	376	219
2	triangular	295	269
3	square	314	261

Surface areas were calculated by taking respective approximations (cylinder, prism and cuboid, respectively) into account as well as that one side is surface-bound and accessible for diffusion.

Surface area of round gel $= \pi r^2 + 2 \pi r h$ with $r = 188$ µm and $h = 219$ µm

$= 0.37 \text{ mm}^2$

Surface area of triangular gel $= \sqrt{3} / 4 a^2 + 3 a h$ with $a = 295$ µm and $h = 269$ µm

$= 0.28 \text{ mm}^2$

Surface area of square gel $= a^2 + 4 a h$ with $a = 314$ µm and $h = 261$ µm

$= 0.43 \text{ mm}^2$

Baylis Hillman reaction between different aldehydes with acrylonitrile in the continuous flow.

a) Synthesis of 2-(hydroxy(2-nitrophenyl)methyl)acrylonitrile. A solution of 2-nitrobenzaldehyde (0.04 mmol) and acrylonitrile (0.08 mmol) was prepared in 1 mL of ethanol:water (v/v = 7:3). The reactant solution was pumped through the reactor (Figure S2) at the flow rate of 0.5 µL/min at 50 ºC for 8 h. The product was collected in a vial from the MFR (excluding a equilibration time of 3.5 h). The collected solution was diluted with water (three folds). The product was extracted with ethyl acetate and the organic layer was concentrated under reduced pressure to yield an orange colour solid, *2*-(hydroxy(*2*-nitrophenyl)methyl) acrylonitrile.

^{1}H NMR (700 MHz, DMSO-d_6, 298 K) δ (ppm) = 8.41 (d, 1H), 8.29 (d, 2H), 8.14 (d, 1H), 7.67 (s, 2H), 6.40 - 6.39 (m, 1H), 6.34 (s, 1H), 5.63 (s, 1H), 5.38 (s, 1H).

ESI-MS (m/z): $C_{10}H_7N_2O_3^+$ [M]$^+$, mass calculated: 203.0457 Da, mass found: 203.0457 Da.

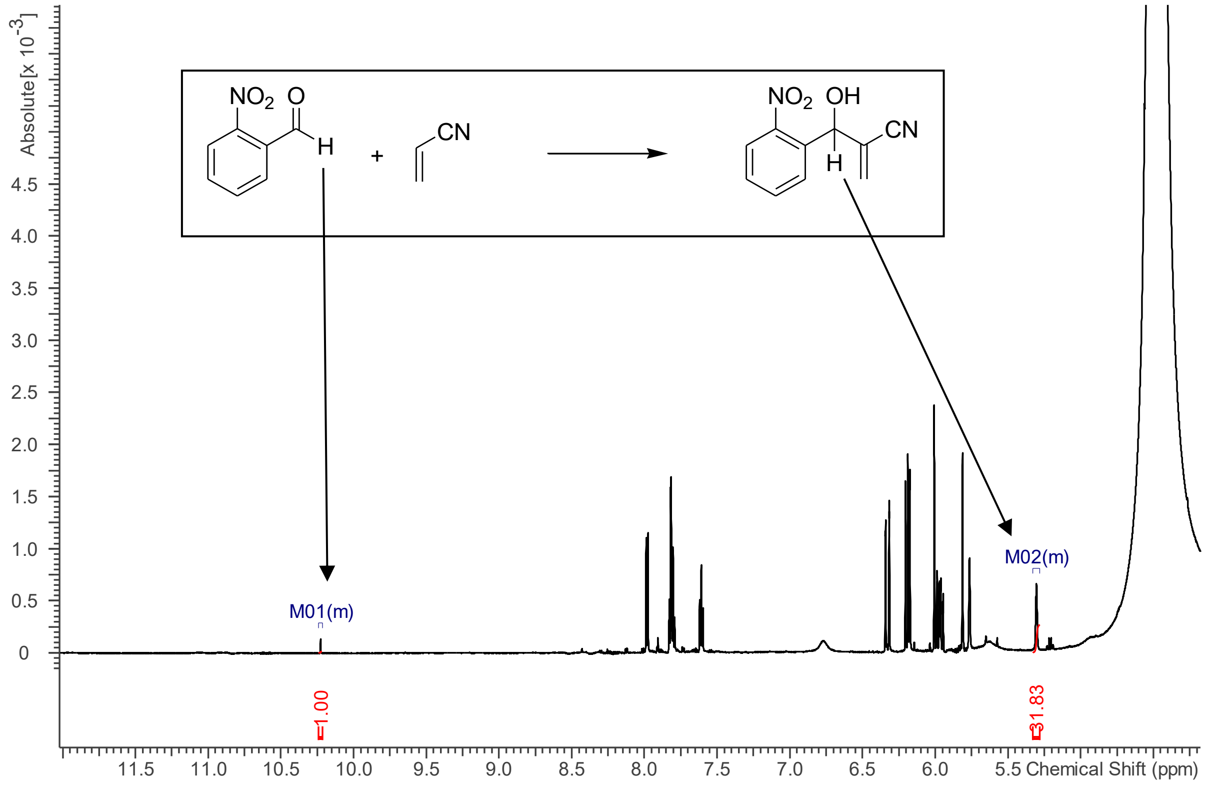

Figure S4. ^{1}H NMR spectra of microfluidic synthesis of *2*-(hydroxy(*2*-nitrophenyl)methyl) acrylonitrile

b) Synthesis of 2-(hydroxy(3-nitrophenyl)methyl)acrylonitrile. A solution of 3-nitrobenzaldehyde (0.04 mmol) and acrylonitrile (0.08 mmol) was prepared in 1 mL of ethanol:water (v/v = 7:3). The reactant solution was pumped through the reactor (Figure S2) at the flow rate of 0.5 µL/min at 50 ºC for 8 h. The product was collected in a vial from the MFR (excluding a equilibration time of 3.5 h). The collected solution was diluted with water (three folds). The product was extracted with ethyl acetate and the organic layer was concentrated under reduced pressure to yield a yellow colour solid, *2*-(hydroxy(*3*-nitrophenyl)methyl)acrylonitrile.

^{1}H NMR (700 MHz, *DMSO-d*$_6$, 298 K) δ (ppm) = 8.26 (t, Hz, 1H), 8.22 - 8.18 (m, 1H), 7.87 - 7.82 (m, 1H), 7.76 - 7.68 (m, 2H), 6.67 (d, 1H), 6.34 - 6.31 (m, 1H), 6.20 (s, 1H), 5.55 (d, 1H).

ESI-MS (m/z): $C_{10}H_7N_2O_3^+$ [M]$^+$, mass calculated: 203.0457 Da, mass found: 203.0457 Da.

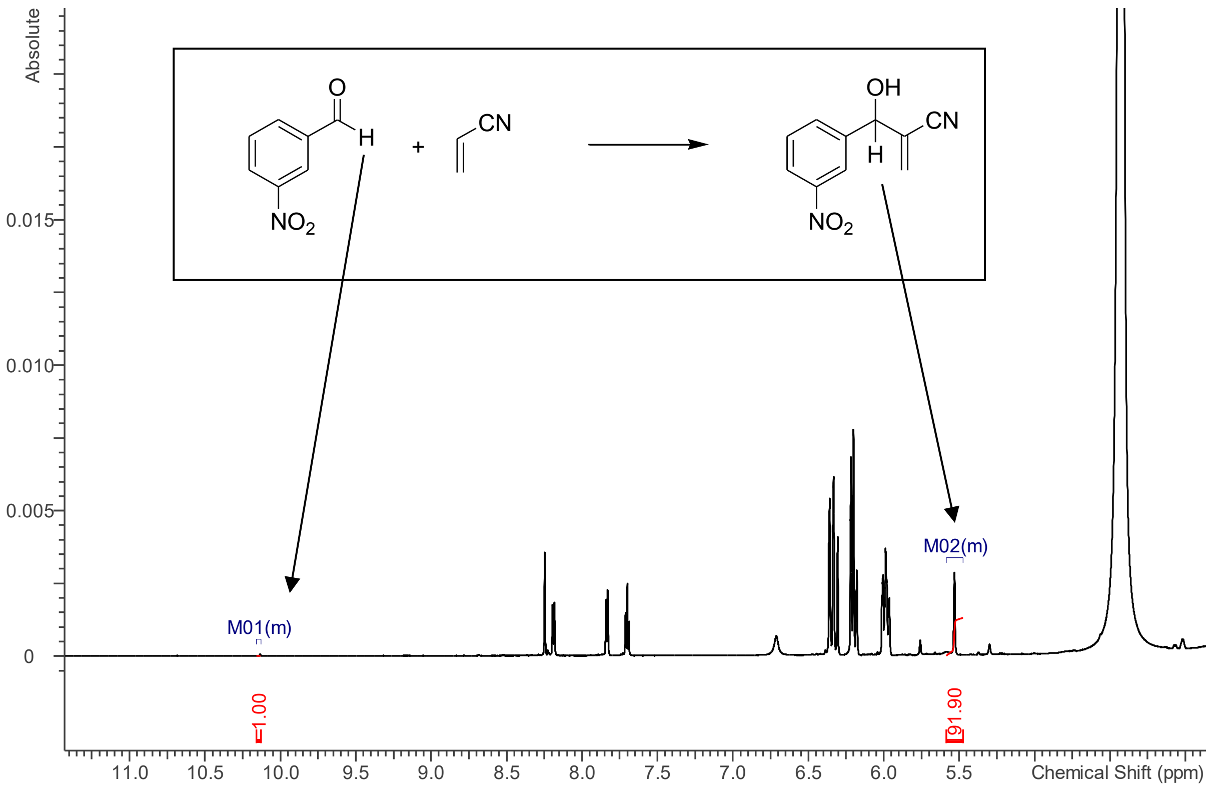

Figure S5. ^{1}H NMR spectra of microfluidic synthesis of *2*-(hydroxy(*3*-nitrophenyl)methyl) acrylonitrile

c) Synthesis of 2-(hydroxy(4-nitrophenyl)methyl)acrylonitrile. A solution of 4-nitrobenzaldehyde (0.04 mmol) and acrylonitrile (0.08 mmol) was prepared in 1 mL of ethanol:water (v/v = 7:3). The reactant solution was pumped through the reactor (Figure S2) at the flow rate of 0.5 µL/min at 50 ºC for 8 h. The product was collected in a vial from the MFR

(excluding a equilibration time of 3.5 h). The collected solution was diluted with water (three folds). The product was extracted with ethyl acetate and the organic layer was concentrated under reduced pressure to yield an orange colour solid, *2*-(hydroxy(*2*-nitrophenyl)methyl)acrylonitrile.

^{1}H NMR (700 MHz, *DMSO-d*$_6$): δ (ppm) = 8.25 (d, 2H), 7.66 (d, 2H), 6.28 (s, 1H), 6.16 (s, 1H), 5.96 - 5.93 (m, 1H), 5.49 (s, 1H).

ESI-MS (m/z): $C_{10}H_7N_2O_3^+$ [M]$^+$, mass calculated: 203.0457 Da, mass found: 203.0453 Da.

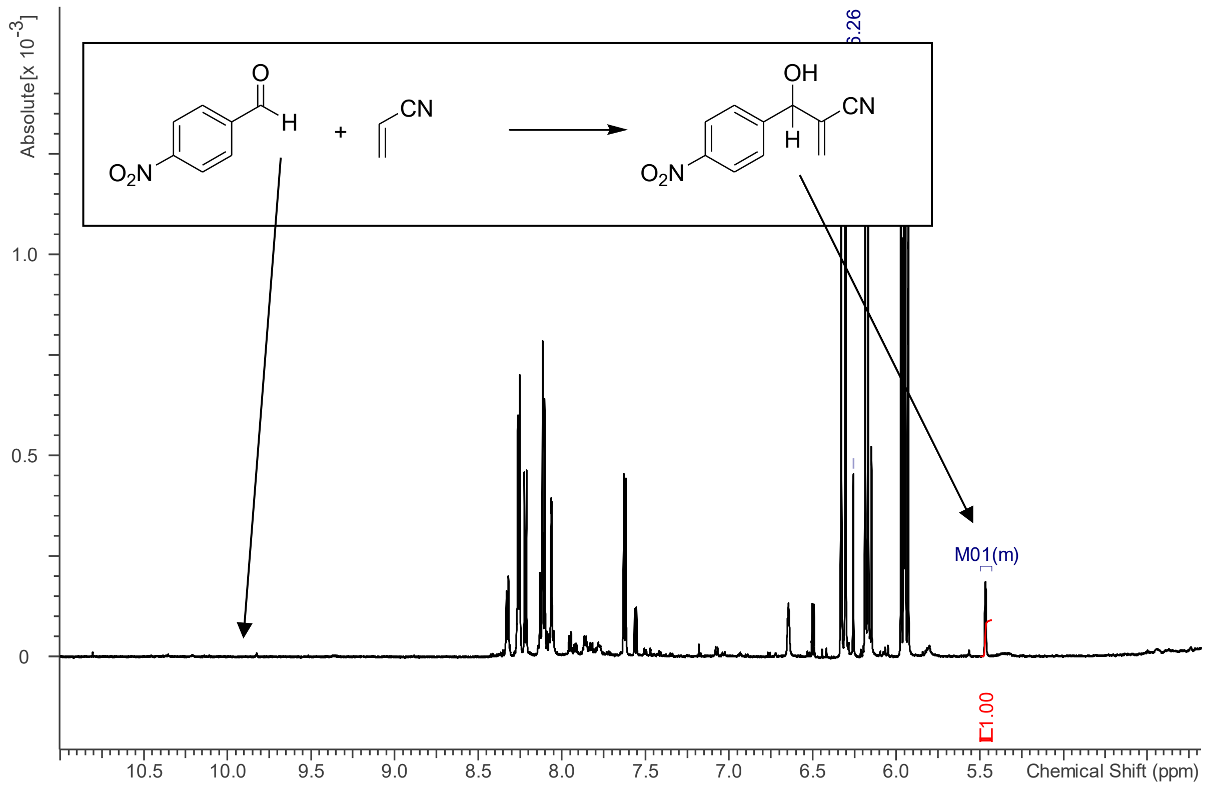

Figure S6. ^{1}H NMR spectra of microfluidic synthesis of *2*-(hydroxy(*4*-nitrophenyl)methyl) acrylonitrile

d) 4-(2-cyano-1-hydroxyallyl)benzonitrile. A solution of 4-formylbenzonitrile (0.04 mmol) and acrylonitrile (0.08 mmol) was prepared in 1 mL of ethanol:water (v/v = 7:3). The reactant solution was pumped through the reactor (Figure S2) at the flow rate of 0.5 µL/min at 50 ºC for 8 h. The product was collected in a vial from the MFR (excluding a equilibration time of 3.5 h). The collected solution was diluted with water (three folds). The product was extracted with ethyl acetate and the organic layer was concentrated under reduced pressure to yield a yellow colour solid, *4*-(*2*-cyano-1-hydroxyallyl)benzonitrile.

^{1}H NMR (700 MHz, *DMSO-d$_6$*): δ (ppm) = 7.87 (d, 2H), 7.59 (d, 2H), 6.57 (d,1H), 6.28 (s, 1H), 6.18 (s, 1H), 5.45 (d, 1H).

ESI-MS (m/z): $C_{11}H_7N_2O^+$ [M]$^+$, mass calculated: 183.0558 Da, mass found: 183.0559 Da.

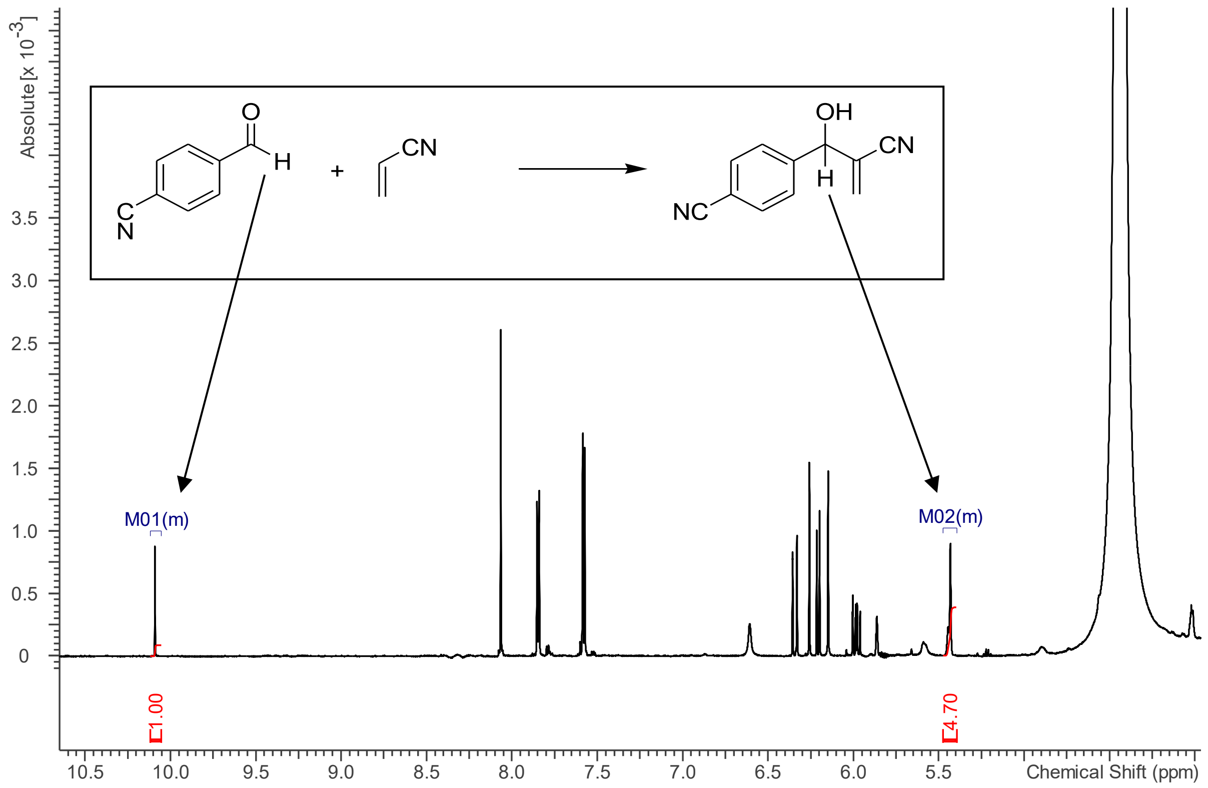

Figure S7. ^{1}H NMR spectra of microfluidic synthesis of *4*-(*2*-cyano-1-hydroxyallyl)benzonitrile

e) 2-((4-fluorophenyl)(hydroxy)methyl)acrylonitrile. A solution of 4-fluorobenzaldehyde (0.04 mmol) and acrylonitrile (0.08 mmol) was prepared in 1 mL of ethanol:water (v/v = 7:3). The reactant solution was pumped through the reactor (Figure S2) at the flow rate of 0.5 µL/min at 50 ºC for 8 h. The product was collected in a vial from the MFR (excluding a equilibration time of 3.5 h). The collected solution was diluted with water (three folds). The product was extracted with ethyl acetate and the organic layer was concentrated under reduced pressure to yield a light yellow colour liquid, *2*-((*4*-fluorophenyl)(hydroxy)methyl)acrylonitrile.

^{1}H NMR (700 MHz, *DMSO-d*$_6$): δ (ppm) = 7.42 (d, 2H), 7.21 (d, 2H), 6.34 (d, 1H), 6.21 (d, 1H), 6.12 (s, 1H), 5.34 (d, 1H).

ESI-MS (m/z): $C_{10}H_7NOF^+$ [M]$^+$, mass calculated: 176.0512 Da, mass found: 176.0520 Da.

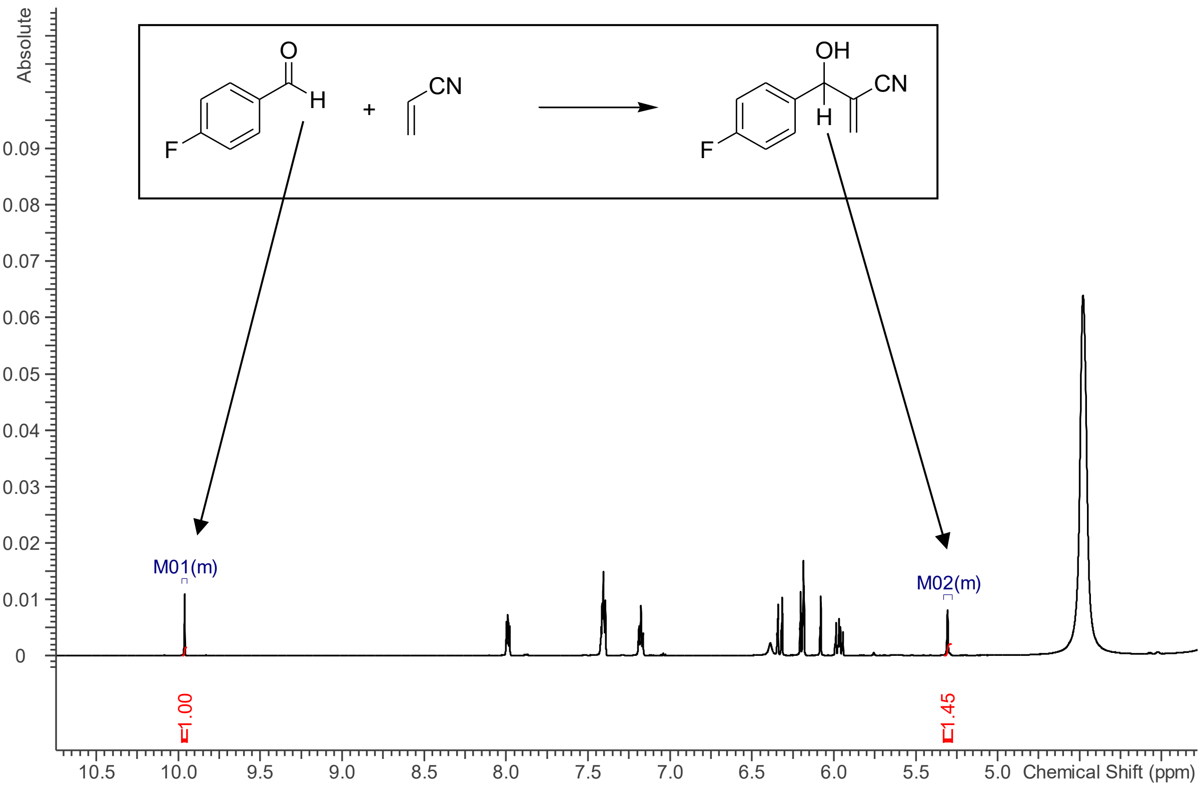

Figure S8. ^{1}H NMR spectra of microfluidic synthesis of *2*-(hydroxy(*4*-fluorophenyl)methyl) acrylonitrile

f) 2-((4-bromophenyl)(hydroxy)methyl)acrylonitrile. A solution of 4-bromobenzaldehyde (0.04 mmol) and acrylonitrile (0.08 mmol) was prepared in 1 mL of ethanol:water (v/v = 7:3). The reactant solution was pumped through the reactor (Figure S2) at the flow rate of 0.5 μL/min at 50 ^{o}C for 8 h. The product was collected in a vial from the MFR (excluding a equilibration time of 3.5 h). The collected solution was diluted with water (three folds). The product was extracted with ethyl acetate and the organic layer was concentrated under reduced pressure to yield a light yellow liquid, *2*-((*4*-bromophenyl)(hydroxy)methyl)acrylonitrile.

^{1}H NMR (700 MHz, *DMSO-d*$_6$): δ (ppm) = 7.62-7.61 (d, 2H), 7.34-7.32 (d, 2H), 6.29 (s, 1H), 6.26 (s, 1H), and 5.16 (s, 1H).

ESI-MS (m/z): $C_{10}H_7NOBr^+$ [M]$^+$, mass calculated: 235.9711 Da, mass found: 235.9708 Da.

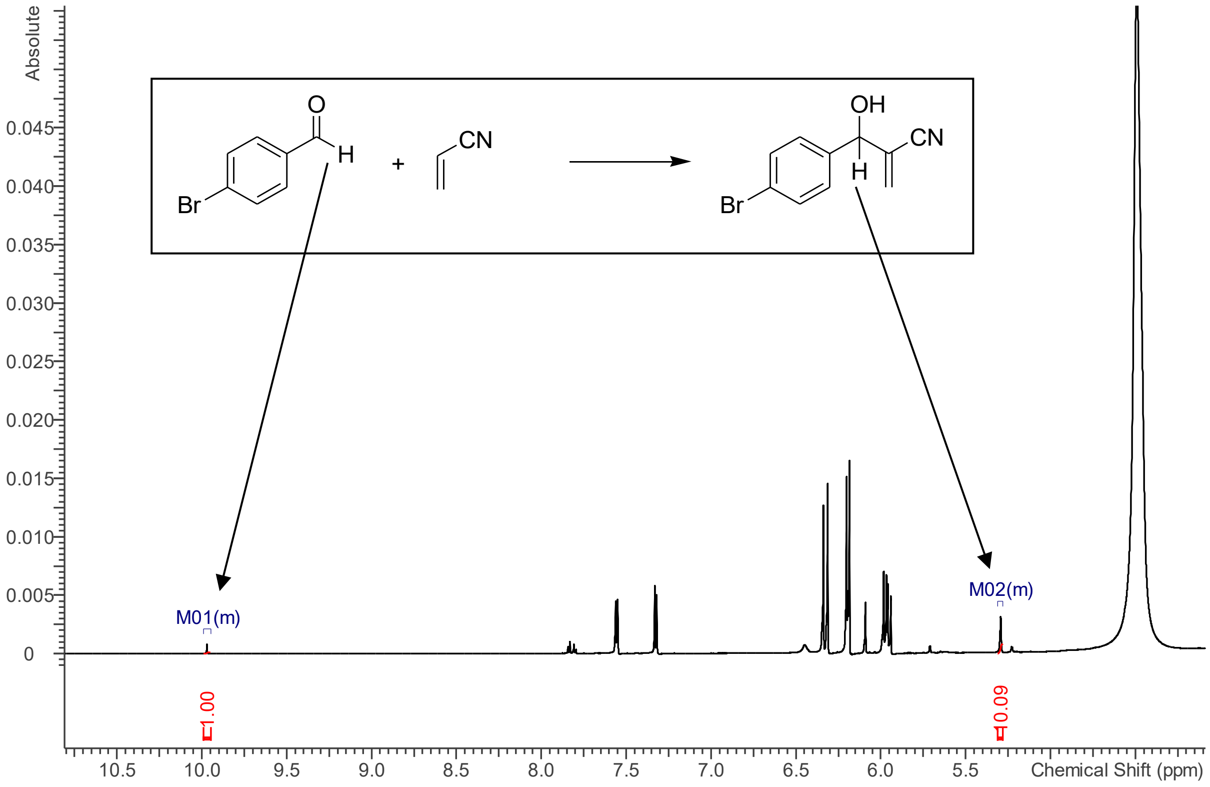

Figure S9. ^{1}H NMR spectra of microfluidic synthesis of *2*-(hydroxy(*4*-bromophenyl)methyl) acrylonitrile

g) 2-((3-chlorophenyl)(hydroxy)methyl)acrylonitrile. A solution of 3-chlorobenzaldehyde (0.04 mmol) and acrylonitrile (0.08 mmol) was prepared in 1 mL of ethanol:water (v/v = 7:3). The reactant solution was pumped through the reactor (Figure S2) at the flow rate of 0.5 µL/min at 50 °C for 8 h. The product was collected in a vial from the MFR (excluding a equilibration time of 3.5 h). The collected solution was diluted with water (three folds). The product was extracted with ethyl acetate and the organic layer was concentrated under reduced pressure to yield a colourless liquid, *2*-((*3*-chlorophenyl)(hydroxy)methyl)acrylonitrile.

^{1}H NMR (700 MHz, *DMSO-d$_6$*): δ (ppm) = 7.40 - 7.36 (m, 1H), 7.36 - 7.34 (m, 1H), 6.45 (s, 1H), 6.28 (s, 1H), 6.15 (s, 1H), 5.38 (s, 1H).

ESI-MS (m/z): $C_{10}H_6NOCl^{2+}$ [M]$^{2+}$, mass calculated: 191.0142 Da, mass found: 191.0138 Da.

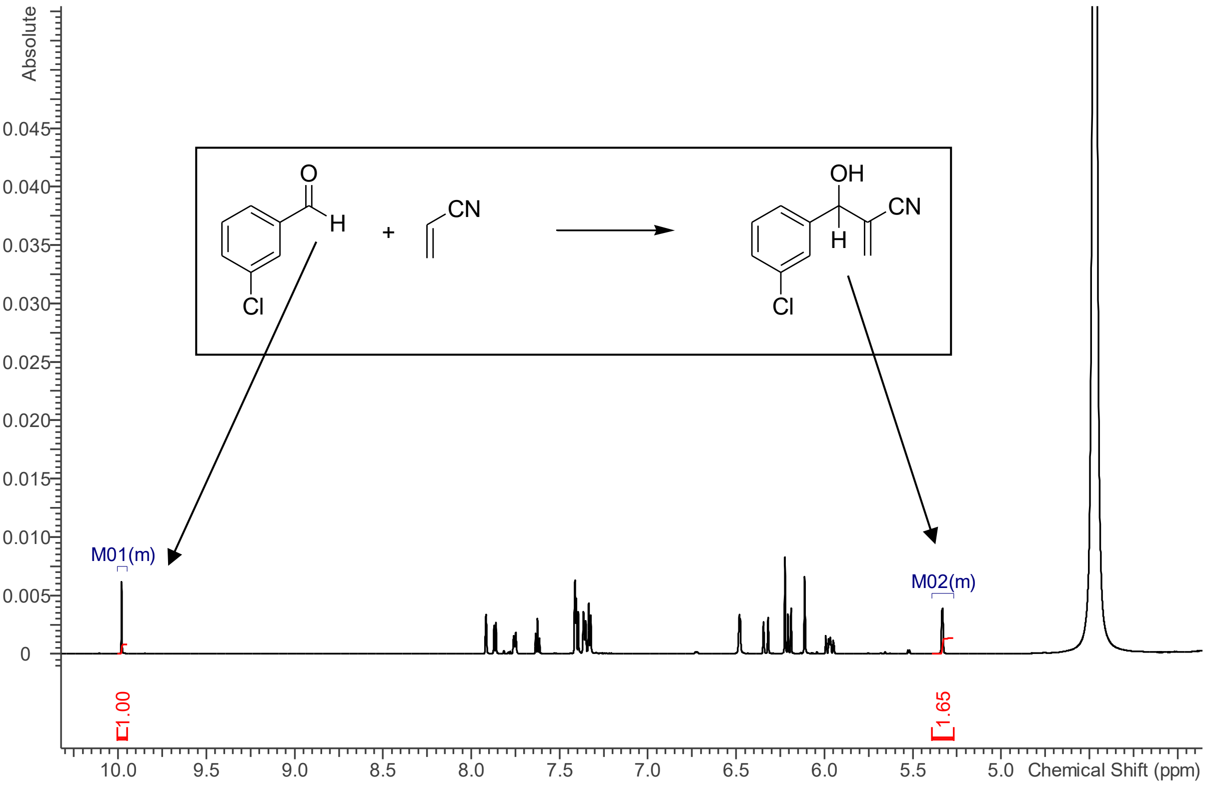

Figure S10. ^{1}H NMR spectra of microfluidic synthesis of *2*-(hydroxy(*3*-chlorophenyl)methyl) acrylonitrile

h) 2-(hydroxy(phenyl)methyl)acrylonitrile. A solution of benzaldehyde (0.04 mmol) and acrylonitrile (0.08 mmol) was prepared in 1 mL of ethanol:water (v/v = 7:3). The reactant solution was pumped through the reactor (Figure S2) at the flow rate of 0.5 µL/min at 50 ºC for 8 h. The product was collected in a vial from the MFR (excluding a equilibration time of 3.5 h). The collected solution was diluted with water (three folds). The product was extracted with ethyl acetate and the organic layer was concentrated under reduced pressure to yield an orange colour solid, *2*-(hydroxy(phenyl)methyl)acrylonitrile.

^{1}H NMR (700 MHz, *DMSO-d*$_6$): δ (ppm) = 7.31 (m, 5H), 6.09 (d, 1H), 6.05 (dd, 1H), 5.61 (s, 1H), 5.39 (s, 1H).

ESI-MS (m/z): $C_{10}H_8NO^+$ [M]$^+$, mass calculated: 158.0700 Da, mass found: 158.0712 Da.

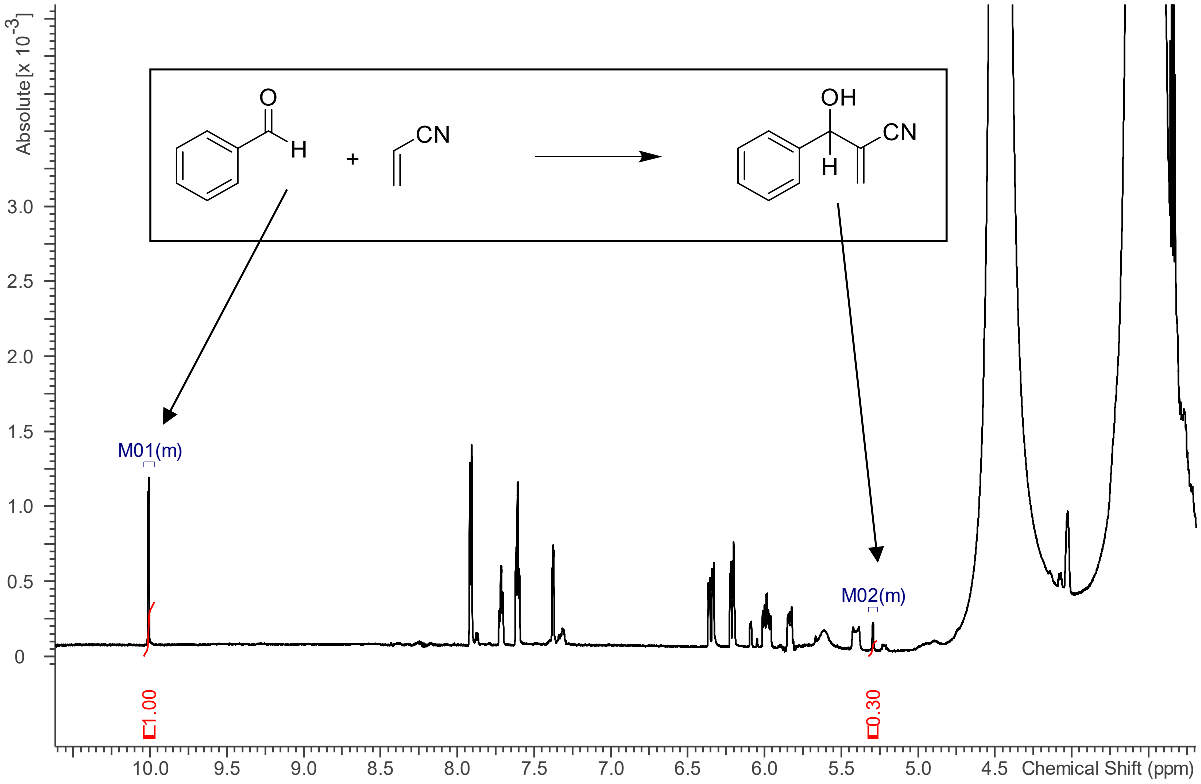

Figure S11. ^{1}H NMR spectra of microfluidic synthesis of *2*-(hydroxy(phenyl)methyl)acrylonitrile

i) 2-(hydroxy(4-methoxyphenyl)methyl)acrylonitrile. A solution of 2-methoxy benzaldehyde (0.04 mmol) and acrylonitrile (0.08 mmol) was prepared in 1 mL of ethanol:water (v/v = 7:3). The reactant solution was pumped through the reactor (Figure S2) at the flow rate of 0.5 µL/min at 50 °C for 8 h. The product was collected in a vial from the MFR (excluding a equilibration time of 3.5 h). The collected solution was diluted with water (three folds). The product was extracted with ethyl acetate and the organic layer was concentrated under reduced pressure to yield a colourless liquid, *2*-(hydroxy(*4*-methoxyphenyl)methyl)acrylonitrile.

^{1}H NMR (700 MHz, *DMSO-d*$_6$): δ (ppm) = 7.28 (d, 3H), 6.96 (d, 2H), 6.21 (s, 1H), 6.19 (s, 1H), 5.06 (s, 1H), 3.76 (s, 3H).

ESI-MS (m/z): $C_{11}H_{10}NO_2^+$ [M]$^+$, mass calculated: 188.0712 Da, mass found: 188.0706 Da.

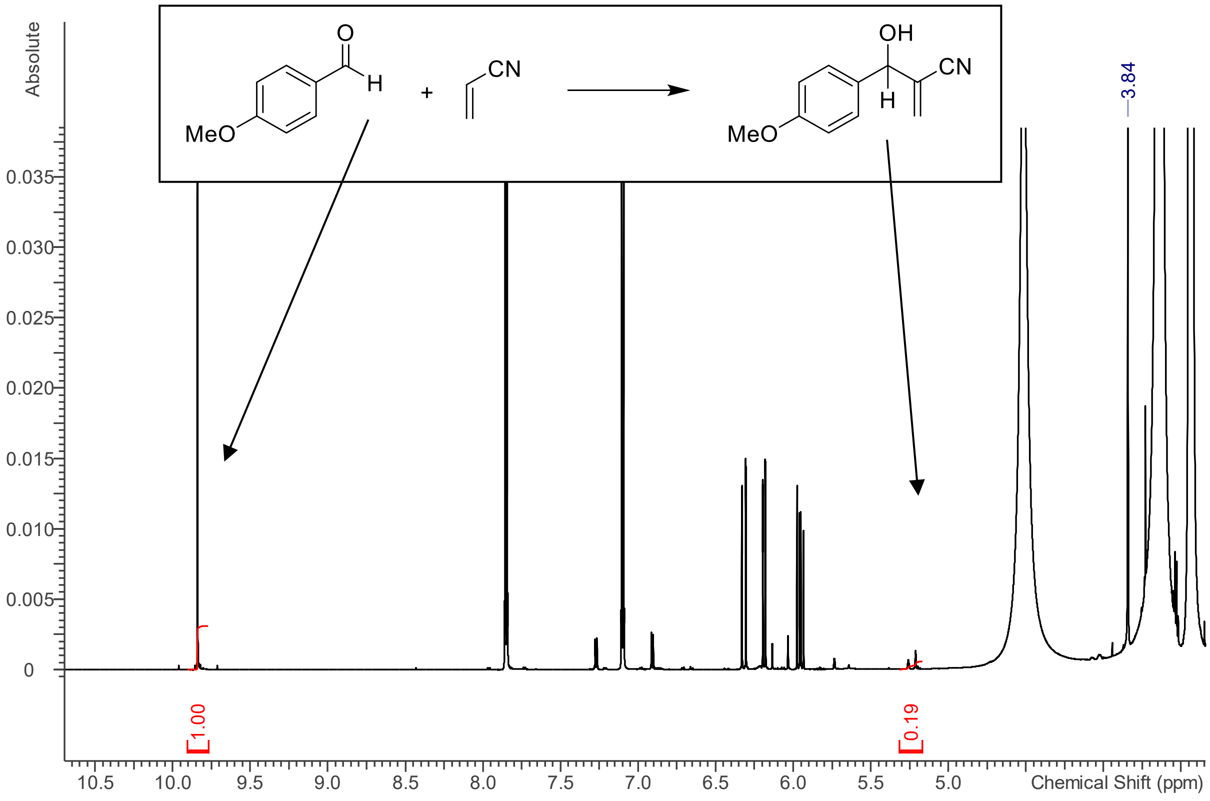

Figure S12. ^{1}H NMR spectra of microfluidic synthesis of *2*-(hydroxy(*4*-methoxyphenyl)methyl) acrylonitrile
